# Dichloridobis(2-chloro­benz­yl)tin(IV)

**DOI:** 10.1107/S1600536810028072

**Published:** 2010-07-17

**Authors:** Kong Mun Lo, Seik Weng Ng

**Affiliations:** a Department of Chemistry, University of Malaya, 50603 Kuala Lumpur, Malaysia

## Abstract

Mol­ecules of the title compound, [Sn(C_7_H_6_Cl)_2_Cl_2_], lie on a twofold rotation axis which passes through the Sn atom. The Sn^IV^ atom exists in a distorted tetra­hedral geometry. Adjacent mol­ecules are linked by weak Sn⋯Cl contacts [3.703 (1) Å], forming a linear chain motif extending along the *b* axis.

## Related literature

For the synthesis of the title compound, see: Sisido *et al.* (1961 ▶). For the crystal structure of dichloridobis(2-fluoro­benz­yl)tin(IV), see: Yin & Gao (2006 ▶).
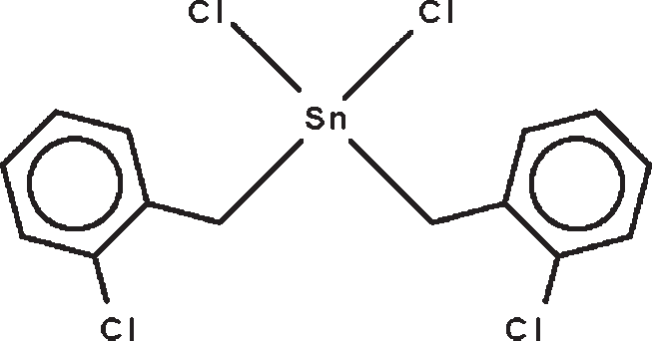


## Experimental

### 

#### Crystal data

[Sn(C_7_H_6_Cl)_2_Cl_2_]
*M* *_r_* = 440.73
Monoclinic, 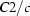

*a* = 26.0750 (13) Å
*b* = 4.7757 (2) Å
*c* = 13.3389 (7) Å
β = 112.1538 (5)°
*V* = 1538.42 (13) Å^3^
*Z* = 4
Mo *K*α radiation
μ = 2.34 mm^−1^
*T* = 100 K
0.40 × 0.10 × 0.10 mm

#### Data collection

Bruker SMART APEX diffractometer
Absorption correction: multi-scan (*SADABS*; Sheldrick, 1996 ▶) *T*_min_ = 0.455, *T*_max_ = 0.800
8736 measured reflections
1767 independent reflections
1674 reflections with *I* > 2σ(*I*)
*R*_int_ = 0.029

#### Refinement

*R*[*F* ^2^ > 2σ(*F* ^2^)] = 0.027
*wR*(*F* ^2^) = 0.073
*S* = 1.07
1767 reflections
87 parameters
H-atom parameters constrained
Δρ_max_ = 2.12 e Å^−3^
Δρ_min_ = −1.03 e Å^−3^

### 

Data collection: *APEX2* (Bruker, 2009 ▶); cell refinement: *SAINT* (Bruker, 2009 ▶); data reduction: *SAINT*; program(s) used to solve structure: *SHELXS97* (Sheldrick, 2008 ▶); program(s) used to refine structure: *SHELXL97* (Sheldrick, 2008 ▶); molecular graphics: *X-SEED* (Barbour, 2001 ▶); software used to prepare material for publication: *publCIF* (Westrip, 2010 ▶).

## Supplementary Material

Crystal structure: contains datablocks global, I. DOI: 10.1107/S1600536810028072/bt5298sup1.cif

Structure factors: contains datablocks I. DOI: 10.1107/S1600536810028072/bt5298Isup2.hkl

Additional supplementary materials:  crystallographic information; 3D view; checkCIF report

## Figures and Tables

**Fig. 1.**
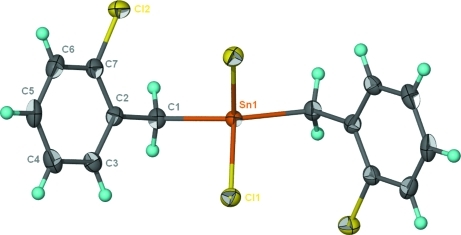
Anisotropic displacement ellipsoid plot (Barbour, 2001) of SnCl2(C7H6Cl)2 at the 70% probability level. Hydrogen atoms are drawn as spheres of arbitrary radius.

## supplementary crystallographic information

### Comment

Diorganotin(IV) dichlorides have the tin centres in a tetrahedral environment but the coordination number can raise by tin–chlorine bridging; the bridging interaction can be regarded as a formal coordination bond if the distance is sufficiently short. In di(2-chlorobenzyl)tin dichloride (Scheme I, Fig. 1), as the interaction is 3.703 (1) Å, the geometry is better interpreted as being tetrahedral. The compound is isostructural with the fluorine analog (Yin & Gao, 2006).

### Experimental

The compound was synthesized by the reaction of metallic tin with 2-benzyl chloride (Sisido *et al.*, 1961), and crystals were obtained by recrystallization from chloroform.

### Refinement

Hydrogen atoms were placed in calculated positions (C–H 0.95–0.99 Å) and included in the refinement in the riding model approximation, with *U*(H) set to 1.2*U*_eq_(C). In the final difference Fouier map there is a peak (2.122e/Å^3^) at  0.96Å from Sn1 and a hole (-1.027e/Å^3^) at 0.79Å from Sn1.

### Crystal data

**Table:**

[Sn(C_7_H_6_Cl)_2_Cl_2_]	*F*(000) = 856
*M**_r_* = 440.73	*D*_x_ = 1.903 Mg m^−^^3^
Monoclinic, *C*2/*c*	Mo *K*α radiation, λ = 0.71073 Å
Hall symbol: -C 2yc	Cell parameters from 7518 reflections
*a* = 26.0750 (13) Å	θ = 3.1–28.3°
*b* = 4.7757 (2) Å	µ = 2.34 mm^−^^1^
*c* = 13.3389 (7) Å	*T* = 100 K
β = 112.1538 (5)°	Block, colorless
*V* = 1538.42 (13) Å^3^	0.40 × 0.10 × 0.10 mm
*Z* = 4	

### Data collection

**Table:**

Bruker SMART APEX diffractometer	1767 independent reflections
Radiation source: fine-focus sealed tube	1674 reflections with *I* > 2σ(*I*)
graphite	*R*_int_ = 0.029
ω scans	θ_max_ = 27.5°, θ_min_ = 1.7°
Absorption correction: multi-scan (*SADABS*; Sheldrick, 1996)	*h* = −33→33
*T*_min_ = 0.455, *T*_max_ = 0.800	*k* = −6→6
8736 measured reflections	*l* = −17→17

### Refinement

**Table:**

Refinement on *F*^2^	Primary atom site location: structure-invariant direct methods
Least-squares matrix: full	Secondary atom site location: difference Fourier map
*R*[*F*^2^ > 2σ(*F*^2^)] = 0.027	Hydrogen site location: inferred from neighbouring sites
*wR*(*F*^2^) = 0.073	H-atom parameters constrained
*S* = 1.07	*w* = 1/[σ^2^(*F*_o_^2^) + (0.0414*P*)^2^ + 6.0977*P*] where *P* = (*F*_o_^2^ + 2*F*_c_^2^)/3
1767 reflections	(Δ/σ)_max_ = 0.001
87 parameters	Δρ_max_ = 2.12 e Å^−^^3^
0 restraints	Δρ_min_ = −1.03 e Å^−^^3^

### Fractional atomic coordinates and isotropic or equivalent isotropic displacement parameters (Å^2^)

**Table:**

	*x*	*y*	*z*	*U*_iso_*/*U*_eq_	
Sn1	0.5000	0.53623 (5)	0.7500	0.01430 (10)	
Cl1	0.53371 (3)	0.85923 (15)	0.89559 (5)	0.02030 (16)	
Cl2	0.33923 (3)	0.48153 (16)	0.55119 (6)	0.02400 (17)	
C1	0.42929 (11)	0.3670 (6)	0.7750 (2)	0.0181 (5)	
H1A	0.4121	0.2202	0.7202	0.022*	
H1B	0.4417	0.2783	0.8473	0.022*	
C2	0.38724 (11)	0.5857 (6)	0.7673 (2)	0.0161 (5)	
C3	0.38871 (11)	0.7290 (6)	0.8594 (2)	0.0192 (5)	
H3	0.4171	0.6866	0.9273	0.023*	
C4	0.34985 (13)	0.9316 (7)	0.8546 (3)	0.0237 (6)	
H4	0.3516	1.0249	0.9187	0.028*	
C5	0.30839 (13)	0.9987 (6)	0.7564 (3)	0.0249 (6)	
H5	0.2820	1.1390	0.7531	0.030*	
C6	0.30554 (12)	0.8602 (7)	0.6627 (3)	0.0232 (6)	
H6	0.2772	0.9050	0.5950	0.028*	
C7	0.34455 (11)	0.6557 (6)	0.6692 (2)	0.0186 (5)	

### Atomic displacement parameters (Å^2^)

**Table:**

	*U*^11^	*U*^22^	*U*^33^	*U*^12^	*U*^13^	*U*^23^
Sn1	0.01278 (15)	0.01420 (15)	0.01702 (15)	0.000	0.00688 (10)	0.000
Cl1	0.0189 (3)	0.0207 (4)	0.0207 (3)	−0.0001 (2)	0.0067 (2)	−0.0046 (2)
Cl2	0.0218 (3)	0.0308 (4)	0.0187 (3)	−0.0035 (3)	0.0068 (3)	−0.0029 (3)
C1	0.0158 (12)	0.0176 (13)	0.0233 (13)	0.0000 (10)	0.0100 (10)	0.0018 (10)
C2	0.0138 (12)	0.0148 (12)	0.0212 (13)	−0.0009 (9)	0.0084 (10)	0.0020 (10)
C3	0.0188 (12)	0.0217 (14)	0.0198 (13)	−0.0020 (10)	0.0104 (10)	0.0023 (11)
C4	0.0258 (15)	0.0219 (15)	0.0304 (15)	−0.0041 (11)	0.0185 (13)	−0.0043 (12)
C5	0.0184 (14)	0.0220 (14)	0.0406 (18)	0.0019 (10)	0.0182 (13)	0.0013 (12)
C6	0.0148 (12)	0.0244 (15)	0.0298 (15)	0.0001 (11)	0.0077 (11)	0.0055 (12)
C7	0.0162 (12)	0.0213 (14)	0.0205 (13)	−0.0025 (10)	0.0093 (10)	−0.0002 (10)

### Geometric parameters (Å, °)

**Table:**

Sn1—C1^i^	2.151 (3)	C2—C7	1.401 (4)
Sn1—C1	2.151 (3)	C3—C4	1.385 (4)
Sn1—Cl1	2.3740 (7)	C3—H3	0.9500
Sn1—Cl1^i^	2.3740 (7)	C4—C5	1.385 (5)
Cl2—C7	1.739 (3)	C4—H4	0.9500
C1—C2	1.489 (4)	C5—C6	1.391 (5)
C1—H1A	0.9900	C5—H5	0.9500
C1—H1B	0.9900	C6—C7	1.389 (4)
C2—C3	1.395 (4)	C6—H6	0.9500
			
C1^i^—Sn1—C1	135.86 (16)	C4—C3—C2	121.7 (3)
C1^i^—Sn1—Cl1	107.23 (8)	C4—C3—H3	119.2
C1—Sn1—Cl1	101.07 (8)	C2—C3—H3	119.2
C1^i^—Sn1—Cl1^i^	101.07 (8)	C5—C4—C3	120.2 (3)
C1—Sn1—Cl1^i^	107.23 (8)	C5—C4—H4	119.9
Cl1—Sn1—Cl1^i^	98.96 (4)	C3—C4—H4	119.9
C2—C1—Sn1	112.17 (18)	C4—C5—C6	119.8 (3)
C2—C1—H1A	109.2	C4—C5—H5	120.1
Sn1—C1—H1A	109.2	C6—C5—H5	120.1
C2—C1—H1B	109.2	C7—C6—C5	119.2 (3)
Sn1—C1—H1B	109.2	C7—C6—H6	120.4
H1A—C1—H1B	107.9	C5—C6—H6	120.4
C3—C2—C7	117.0 (3)	C6—C7—C2	122.1 (3)
C3—C2—C1	120.5 (2)	C6—C7—Cl2	118.2 (2)
C7—C2—C1	122.5 (3)	C2—C7—Cl2	119.7 (2)

Symmetry codes: (i) −*x*+1, *y*, −*z*+3/2.
